# The Role of Emotion Regulation in Reducing Emotional Distortions of Duration Perception

**DOI:** 10.3389/fpsyg.2018.00347

**Published:** 2018-03-15

**Authors:** Yu Tian, Peiduo Liu, Xiting Huang

**Affiliations:** Faculty of Psychology, Southwest University, Chongqing, China

**Keywords:** duration perception, emotion regulation, reappraisal, suppression, emotion

## Abstract

Emotional events, especially negative ones, are consistently reported to last longer than neutral events. Previous studies suggested that this distortion of duration perception is linked to arousal and attention bias in response to emotional events. Reappraisal and suppression, arguably the most effective strategies for emotion regulation, have been demonstrated to decrease such arousal and attention bias. The present study investigated whether reappraisal and suppression can reduce emotional distortions of duration perception. Seventy-eight Chinese undergraduates were recruited as paid participants and randomly assigned to nonregulation, reappraisal, and suppression groups. Before they performed a temporal bisection task involving presentation of emotional pictures for different durations, the groups were each given one of three different sets of instructions requiring them to passively perceive, reappraise, or suppress the emotions of the pictures. The results indicated that the distortion of duration perception occurred only in the nonregulation group, suggesting that it can be effectively reduced by reappraisal and suppression.

## Introduction

Duration perception refers to one’s perception of the interval between two successive events or the duration of an event (Fraisse, 1984). The perception of duration is essential for both survival and the completion of everyday activities (Zhang et al., 2014). It can be distorted through numerous factors, such as age, mental status, and sensory channel. Among these factors, emotion might be one of the strongest and most salient (Lake, 2016). Numerous studies have shown that emotions are associated with distortions of duration perception (Gan et al., 2009; Droit-Volet et al., 2010; Gil and Droit-Volet, 2011; Droit-Volet, 2012). The temporal distortion is believed to reflect that duration perception is modulated by changes in the environment (Lake et al., 2016), allowing people to adaptively respond to stimuli in their environment (e.g., Harrington et al., 2011; Lewis and Meck, 2012; Matthews and Meck, 2014). Nevertheless, in some situations, people might desire a reduction of this emotional distortion of duration perception. For example, on the football field, when the goalkeeper sees an aggressive striker, a distorted duration perception might interfere with when they decide to catch the ball; while driving, a distorted duration perception would interfere with a driver’s decision on when to stop upon seeing a hazard in their path. Therefore, it might be necessary to explore the ways of reducing emotional distortions of duration perception.

The psychological mechanism of the emotional distortion of duration perception is typically explained using the pacemaker–accumulator (PA) model (Gibbon et al., 1984). The PA model consists of three sequential stages: clock, memory, and decision-making. During the clock stage, a pacemaker emits pulses through a switch, and these pulses are then collected by an accumulator. The number of collected pulses represents the duration. In the memory stage, the duration (represented by the clock stage) is stored in working memory, and an appropriate reference memory for that duration is retrieved from long-term memory. Finally, in the decision-making stage, the duration being timed is compared to the reference memory to determine if the duration is shorter, longer, or equal. Although variations in each part of this model might distort duration perception, researchers have attached particular importance to the impulse rate and switch, which are two of the most salient mechanisms in the PA model (Lake et al., 2016; Wang et al., 2016). An accelerating impulse rate is in general associated with increasing arousal. More specifically, an accelerating impulse rate would lead to a greater number of pulses collected by the accumulator, resulting in an overestimated duration perception (Droit-Volet et al., 2013; Schwarz et al., 2013; Volkinburg and Balsam, 2014; Yoo and Lee, 2015). However, recent studies have shown that the relation between impulse rate and arousal is not as direct as previously thought (see Burle and Casini, 2001), as it is moderated by the emotional state of the individual (Mella et al., 2011), stimulus content (Gil and Droit-Volet, 2012), and the duration of stimulus presentation (Fayolle et al., 2015). On the other hand, the switch is thought to reflect attention allocation, which in this context is defined as the allocation of processing resources to timing versus other processes (Zakay and Block, 1996; Ferrara et al., 1997; Lejeune, 1998; Mella et al., 2011). The switch regulates the flow of pulses into the accumulator based on an open and closed state, and the allocation of time-relevant attention determines the perceived duration (Zakay, 2005; Brosch et al., 2013; Séguin, 2013; Block and Gruber, 2014). When less attention is allocated to timing, the switch closes at a less frequent rate, meaning that fewer pulses are allowed to pass into the accumulator. In contrast, when more attention is allocated to timing, the switch closes more frequently, meaning that more pulses are allowed to pass through.

According to numerous studies, the duration of emotional stimuli tends to be perceived as longer than that of neutral stimuli. Particularly, duration has been found to be overestimated for emotional faces (Gil and Droit-Volet, 2011; Yoo and Lee, 2015; Ishikawa and Okubo, 2016), emotional scene images (Angrilli et al., 1997), emotional sounds (Noulhiane et al., 2007; Zhang et al., 2012), and emotional films (Droit-Volet et al., 2011). Previous studies have proposed that emotions influence arousal, which speeds up the internal clock (Volkinburg and Balsam, 2014; Yoo and Lee, 2015). Some studies have also suggested that attention bias to emotional stimuli makes the switch close earlier compared with neutral stimuli (Lui et al., 2011; Smith et al., 2011). Consequently, both arousal and attention have been suggested as mechanisms for the emotional distortion of duration perception (Droit-Volet, 2013b; Lake et al., 2016).

There is some evidence that emotional distortions in duration perception can be modified. Effron et al. (2006) found that participants who held a pen in their mouths in order to suppress their expressions no longer overestimated the presentation duration of emotional faces relative to neutral faces. Mella et al. (2011) used prior instructions to manipulate participants to attend to time, emotion, or both, and found that participants overestimated the duration of highly negative stimuli when they attended to emotion compared to when they attended to time or divided their attention between time and emotion. Droit-Volet et al. (2015) found the awareness of emotion-related distortions in duration perception modify the emotional distortion: when the participants were told either that the perception of emotional stimuli produces an overestimation of duration (true) or that it produces an underestimation (false), the effects of emotional stimuli on duration perception were amplified or reduced, respectively. Taken together, this evidence suggests that people can regulate the emotional distortion of duration perception.

There is much literature on emotion regulation showing that emotion can be influenced in an adaptive way (see Gross, 1998, 2007, 2014). The process model of emotion regulation, proposed by Gross (1998), provides five major emotion regulation strategies that may be used during the emotion production process: situation selection, situation modification, attention allocation, cognitive reappraisal, and expression suppression. Arguably, the most common and effective of these strategies are the latter two: reappraisal and suppression (Cheng et al., 2009; Cai et al., 2016; Chen et al., 2017a). For this reason, reappraisal and suppression were selected as emotion regulation strategies for use in the present study. Reappraisal is an antecedent-focused strategy that occurs at the early stage of emotion production, whereby emotions are regulated by changing one’s understanding of the emotional events. In contrast, suppression is a response-focused strategy that occurs at the late stage of emotion production and involves the regulation of emotion through suppressing its expression. There is empirical evidence that both reappraisal and suppression decrease emotional arousal and modulate emotional attention bias, and can even change brain activity related to emotional processing (Eippert et al., 2007; Goldin et al., 2008; Driscoll et al., 2009; Flynn et al., 2010; Cai et al., 2016; Chen et al., 2017a). Accordingly, they are potential strategies for reducing emotional distortions of duration perception. The present study hypothesizes that reappraisal and suppression are effective for reducing emotional distortions of duration perception.

Reappraisal and suppression are both costly strategies in terms of cognitive resources (Polivy, 1998; Gross and John, 2003; Bonanno et al., 2004; Mauss et al., 2006), and might therefore lead to deficits in attention. Using a dual-task paradigm, Chen et al. (2007) found the less attention allocated to a time-irrelevant task, the lower the perceived duration of that task. This suggests that the use of these emotion regulation strategies might cause people to underestimate the perceived duration of a task or event. However, the costs of reappraisal and suppression are different, with suppression being regarded as costlier than reappraisal (e.g., Richards and Gross, 2000; Goldin et al., 2008). A similar phenomenon was observed with East-Asian participants, who are believed to be particularly good at using suppression to regulate emotion (Yuan et al., 2014). This suggests that suppression might result in less attention being given to time-relevant tasks compared to reappraisal. Therefore, the present study hypothesizes that the perceived duration of stimulus presentation would be shorter when suppression is used as an emotion regulation strategy than when reappraisal is used.

## Materials and Methods

Considering that the perceived duration of negative stimuli tends to be overestimated compared to positive stimuli (Gan et al., 2009; Lake et al., 2016), and most studies of emotion regulation focused on reducing negative emotions (e.g. Aldao et al., 2015; Sheppes et al., 2015; Yoon et al., 2015), negative and neutral pictures were selected as stimuli in this study. To avoid any potential interaction between reappraisal and suppression, each participant was allocated to only one of these experimental conditions. Thus, the experiment adopted a mixed 3 × 2 design consisting of the between-subject factor of group (nonregulation, reappraisal, suppression) and the within-subject factor of picture type (neutral, negative).

### Participants

We determined the sample size according to an *a priori* power analysis and by referencing previous studies. To detect a large emotion regulation effect (Cohen’s *d* = 0.8) with a power of 0.8 at an alpha of 0.05, the power analysis indicated that a sample size of 26 participants per group was appropriate. This sample size was also generally consistent with previous studies on the emotional distortion of duration perception and emotion regulation. Thus, 78 healthy participants (39 females; age range = 21–26 years) were recruited from Southwest University in China as paid participants. Participants were randomly assigned to one of three groups: nonregulation (12 female and 14 male), reappraisal (13 female and 13 male), and suppression (14 female and 12 male). All participants were right-handed and had normal or corrected-to-normal vision. They gave their written informed consent prior to the experiment. The experimental protocol was approved by the local ethics committee of Southwest University.

To avoid the potential influence of anxiety, depression, and neuroticism on duration perception, all participants were tested by Spielberger State-Trait Anxiety Inventory (Spielberger, 1970), Beck Depression Inventory (Beck et al., 1996), and NEO Five-Factor Inventory (Costa and McCrae, 1992). The results showed that all participants scored low in anxiety, depression, and neuroticism. Furthermore, the three groups did not show a significant difference in state anxiety, *F*(2,75) = 0.34, *p* > 0.05; trait anxiety, *F*(2,75) = 0.01, *p* > 0.05; depression, *F*(2,75) = 1.37, *p* > 0.05; or neuroticism, *F*(2,75) = 2.47, *p* > 0.05. To avoid the potential influence of emotion regulation ability, all participants completed the Emotion Regulation Questionnaire (ERQ; Gross and John, 2003). The results showed that the three groups did not significantly differ in reappraisal score, *F*(2,75) = 0.83, *p* > 0.05, or suppression score *F*(2,75) = 0.70, *p* > 0.05, see **Table 1**. Taken together, the results of these tests show that the three groups were sufficiently homogenous.

**Table 1:** Mean scores (standard deviation) of anxiety, depression, neuroticism, and emotion regulation ability for nonregulation, suppression, and reappraisal groups.

	Nonregulation	Suppression	Reappraisal
State anxiety	34.15(6.70)	32.85(7.46)	34.50(8.54)
Trait anxiety	38.92(7.33)	39.00(8.06)	39.00(8.89)
Depression	7.73(7.22)	6.88(4.99)	9.92(7.95)
Neuroticism	23.88(7.40)	19.50(6.56)	21.19(7.54)
Reappraisal	30.62(4.87)	31.31(5.45)	31.42(5.12)
Suppression	18.65(4.75)	19.31(4.20)	20.12(4.41)


### Stimuli

The stimuli used for the representation of duration were a brown rectangle image, 16 neutral pictures and 16 negative pictures (size: 433 × 315 pixels). All pictures were selected from the Chinese Affective Picture System (CAPS). Negative pictures depicted scenes featuring frightening animals, dilapidated buildings, and disaster areas, while neutral pictures depicted scenes of neutral animals, buildings, and human activities. Each picture was assessed in a previous survey in terms of its valence and arousal on a nine-point scale in a large sample of Chinese participants (Lu et al., 2005). An independent samples *t*-test performed on the average scores showed that the two categories of negative pictures differed significantly from neutral pictures in valence, *t*(30) = -21.24, *p* < 0.05 (mean ± standard deviation: negative = 2.74 ± 0.32, neutral = 5.26 ± 0.35), and arousal, *t*(30) = 20.00, *p* < 0.05 (negative = 5.78 ± 0.48, neutral = 3.32 ± 0.08).

### Procedures

The experiment was run and participants’ responses recorded using E-Prime 1.1 (Psychological Software Tools, Pittsburgh, PA, United States) on a PC. The stimuli were presented using a 17” LCD monitor (1,024 × 768 pixels, 60-Hz) and the participants made their responses using the computer’s keyboard. The participants were seated in a quiet room approximately 60 cm from the pictures, with horizontal and vertical visual angles of below 16°.

The experiment consisted of two parts: emotion regulation and duration perception. In the emotion regulation part, participants in each group were provided with one of three sets of instructions in writing (nonregulation, reappraisal, or suppression), which they silently deliberated on for 1 min. The nonregulation instruction asked participants to passively perceive the emotions arising in response to the pictures. The reappraisal instruction asked participants to intentionally reappraise that the pictures had been created by a computer program and did not depict true events. Finally, the suppression instruction asked participants to intentionally suppress their expressions when a picture appeared on the screen.

**FIGURE 1:**
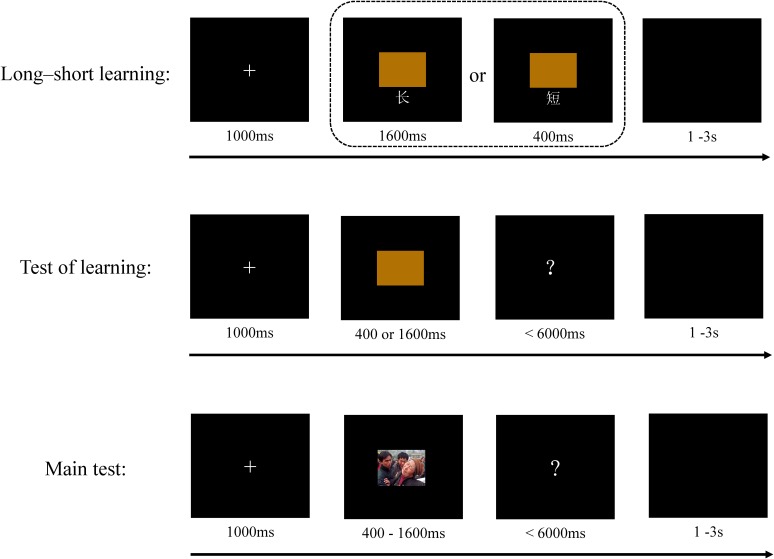
Schematic illustration of the temporal bisection task.

In the duration perception part, the temporal bisection task, which has been used widely in previous studies, was adopted. The procedure was similar to that described in earlier research (Droit-Volet et al., 2004; Tipples, 2008). The participants were initially trained to differentiate “short” (400 ms) from “long” (1,600 ms) stimulus durations. On the first 10 trials, the image of a brown rectangle appeared for a short or a long duration in a random sequence along with a corresponding Chinese character for “short” or “long” marked underneath it. The participants were asked to remember and be ready to differentiate between “short” and “long” durations. Following the training, a test was conducted to confirm that the training had been effective. In the test, the participants were asked to indicate whether the stimulus appeared for either a short or a long stimulus duration by pressing one of two labeled keys (“j” and “f”) on the keyboard. The response mapping (e.g., “j” and “f” for the short and long duration images, respectively) was counterbalanced across the participants. To avoid any potential encouragement- or frustration-induced emotion, no feedback was provided during the test. If a participant’s accuracy did not reach 100%, he/she was trained again. Participants were given a 2-min break between the post-training test and the main test. During the main test, negative and neutral pictures were randomly presented for seven durations (400, 600, 800, 1,000, 1,200, 1,400, and 1,600 ms), see **Figure 1**. Each picture was presented for each duration. Accordingly, 224 trials were conducted. The participants were asked to look at each picture and indicate whether it had appeared for a duration that was closer to either the short or the long duration previously learned. To avoid the possible effects of fatigue on the results, three 1-min breaks were given following trials 56, 112, and 168. Each break was followed by the emotion regulation instruction and a long–short training to consolidate the effectiveness of training. No more tests were conducted to confirm training effect. At the end of the temporal bisection task, the participants were asked to respond to a question – “Did you follow the instructions given to you for judging the duration?” (given here in English, but actually presented in Chinese; the same was true with the next question) on a seven-point scale (from 1 = “not at all” to 7 = “completely”). They were also required to answer the question “How much effort did you exert to regulate (or suppress or reappraise) the emotions arising in you in response to the picture?” on a seven-point scale (from 1 = “not at all” to 7 = “completely”). The words “regulate,” “suppress,” or “reappraise” were presented for the nonregulation, suppression, and reappraisal groups, respectively.

### Statistics

**FIGURE 2:**
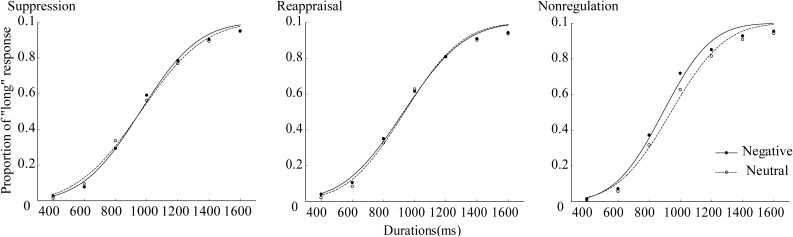
Proportion of “long” response plotted as a function of stimulus duration for suppression, reappraisal, and nonregulation groups.

The temporal bisection task was analyzed by computing the proportion of long duration responses for each stimulus duration. If the stimulus duration was represented as “*t*,” the proportion of long duration responses was designated as *P*(long|*t*). A plot of the proportions formed a psychometric function that was described as a Gaussian cumulative distribution function (*S*) curve. The start of the resulting *S* curve was at approximately zero, representing the shortest durations, and its end was at approximately 1, representing the longest durations. The resulting curves were fitted with a cumulative Gaussian function, with the mean (μ) corresponding to the “Point of Subject Equality” (PSE) and the SD (σ) corresponding to the temporal sensitivity. The PSE was the duration that yielded no difference between long and short responses, that is, *P*(long|*t* = μ) = 0.5 (Krogercosta et al., 2013; Liu et al., 2015). The generalization gradients related to the proportion of “long” responses to stimulus duration were fitted via cumulative Gaussian functions with the following equation:

y =0.5×{1+erf[(x−μ)/(σ×√2)]}

where *y* is the proportion of “long” responses, *x* is the objective duration, and *erf* is the error function or Gauss error function. The proportion of “long” responses was plotted as a function of stimulus duration for the three groups. The lines were the best-fit cumulative Gaussian, see **Figure 2**.

Statistical analysis was performed using SPSS Statistics 20.0 (Armonk, NY, United States). The significance level was set at 0.05. Repeated-measures ANOVAs were performed on the PSE measurements with group (nonregulation, reappraisal, and suppression) and picture type (neutral, negative) as factors. *Post hoc* testing of the significant main effects was conducted using the LSD method. Significant interactions were analyzed using simple effects models. Partial η-squared ($\eta_{p}^{2}$) was reported as a measure of effect size for the ANOVAs, with 0.05, 0.10, and 0.20 indicating a small, medium, and large effect, respectively.

## Results

### Manipulation Check

The first manipulation check aimed to confirm the effect of emotional distortion, which served as the baseline in the analyses on emotion regulation. The data of nonregulation group were used for this analysis. According to a paired samples *t*-test, the PSE of the negative pictures (897.95 ± 127.69 ms) was significantly different from that of the neutral pictures (955.11 ± 142.64 ms), *t*(25) = -3.53, *p* < 0.05, indicating that the manipulation of emotion was effective.

The second manipulation check aimed to determine whether each participant followed the instructions of the temporal bisection task. Using the self-report scores in response to the question, “Did you follow the instructions given to you for judging the duration?” A one-way ANOVA with group as a between-subject variable showed a significant main effect of group, *F*(2,75) = 4.75, *p* < 0.01, $\eta_{p}^{2}$ = 0.11 (nonregulation group = 6.23 ± 0.65, suppression group = 5.69 ± 1.05, reappraisal group = 6.35 ± 0.69). The *post hoc* analysis revealed that the mean score of the suppression group was smaller than those of the nonregulation and reappraisal groups, *p*s < 0.05. There was no difference between the nonregulation and reappraisal groups, *p* = 0.61. In addition, three one-sample *t*-tests were conducted. These revealed that the mean scores for each group were significantly higher than the midpoint of the rating scale (i.e., 4), *t*s(25) = 17.46–8.22, *p*s < 0.05, suggesting that participants followed the instructions of the temporal bisection task, but the scores of the suppression group were not as good as those of the nonregulation and reappraisal groups.

The third manipulation check aimed to determine whether participants followed the emotion regulation instruction. Using participants’ self-report scores in response to the question, “How much effort did you exert to regulate (or suppress, or reappraise) the emotions arising in you in response to the picture?” A one-way ANOVA with group as a between-subject variable showed a significant main effect of group, *F*(2,75) = 11.58, *p* < 0.05, $\eta_{p}^{2}$ = 0.24 (nonregulation group = 3.46 ± 2.12, suppression group = 5.15 ± 1.32, and reappraisal group = 5.50 ± 1.33). The *post hoc* analysis revealed that the mean score of the nonregulation group was smaller than those of the reappraisal and suppression groups, *p*s < 0.05. There was no difference between the reappraisal and suppression groups, *p* = 0.45. In addition, three one-sample *t*-tests were performed to test whether the mean score for each group differed from the midpoint of the rating scale (i.e., 4). The results indicated that the mean score for the nonregulation group was not significantly different from the midpoint of the rating scale, *t*(25) = -1.29, *p* > 0.05. In contrast, the mean rating score for the suppression group was significantly higher than the midpoint, *t*(25) = 4.47, *p* < 0.05, and the mean rating score for the reappraisal group was significantly higher than the midpoint, *t*(25) = 5.73, *p* < 0.05. These results suggested that participants followed the emotion regulation instructions that they were given.

### Effect of Emotion Regulation on Duration Perception

**FIGURE 3:**
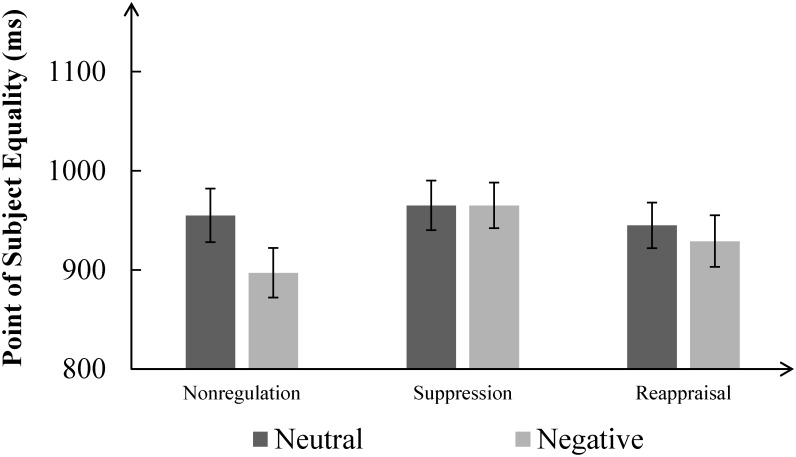
Mean point of subjective equality for neutral pictures and negative pictures in each group. The error bar represents standard error.

A repeated-measures ANOVA of PSE with group (nonregulation, reappraisal, suppression) as a between-subject factor and picture type (neutral, negative) as a within-subject factor showed a non-significant main effect of group, *F*(2,75) = 0.69, *p* = 0.50, a significant main effect of picture type, *F*(2,75) = 6.72, *p* < 0.05, $\eta_{p}^{2}$ = 0.08, and a significant interaction between group and picture type, *F*(2,75) = 3.31, *p* < 0.05, $\eta_{p}^{2}$ = 0.08. A simple effect analysis showed that in the nonregulation group, the PSE of negative pictures (897.95 ± 127.69 ms) was shorter than was that of neutral pictures (955.11 ± 142.64 ms), *p* < 0.05. In the reappraisal group, there was no difference in PSE between negative pictures (929.55 ± 134.07 ms) and neutral pictures (945.46 ± 119.47 ms), *p* = 0.33. Finally, in the suppression group, there was also no difference in PSE between negative pictures (965.43 ± 118.45 ms) and neutral pictures (965.32 ± 130.06 ms), *p* = 0.99. Thus, the emotional distortion was observed only in the nonregulation group, see **Figure 3**. Another simple effect analysis showed there wasn’t significant group effect for neutral pictures (*p* > 0.05). The group effect was only marginally significant between nonregulation group and suppression group (*p* = 0.06), but the group effect between nonregulation group and reappraisal group wasn’t significant (*p* > 0.05).

To avoid the potential influence of different baselines, we used the PSE of neutral pictures and PSE of negative pictures to count the emotional overestimation of each participant (PSE_neutral_ – PSE_negative_ = PSE_overestimated_). A one-way ANOVA which used group (nonregulation, reappraisal, suppression) as between-subject variable showed the effect of group was significant, *F*(2,75) = 3.31, *p* < 0.05, $\eta_{p}^{2}$ = 0.08. The *post hoc* analysis identified the group effect was significant between nonregulation group and suppression group (*p* = 0.02). A marginally significant group effect was observed between nonregulation group and reappraisal group (*p* = 0.07). There was no significant difference between suppression group and reappraisal group (*p* = 0.49).

The results suggested that reappraisal and suppression, which are two typical emotion regulation strategies, can effectively reduce the emotional distortion of duration perception.

## Discussion

The present study employed negative and neutral pictures to examine whether emotion regulation can reduce the emotional distortion of duration perception. More precisely, 78 healthy participants were randomly assigned to nonregulation, reappraisal, and suppression groups, wherein they were told to passively perceive, reappraise, or suppress the stimulus-induced emotions, respectively. Subsequently, they completed a temporal bisection task, which was used to investigate the duration perception of each group (with durations ranging from 400 to 1600 ms).

First, in the nonregulation group, we observed that negative pictures distorted duration perception when compared with neutral stimuli. This confirms that emotion distorts duration perception. This finding is consistent with those of previous studies, which reported that people tended to overestimate the duration of emotional stimuli, especially negative ones, compared with neutral stimuli (Angrilli et al., 1997; Tipples, 2008; Gan et al., 2009; Volkinburg and Balsam, 2014). Negative stimuli typically lead to increased arousal and attention bias (Yiend, 2010; Hoffman et al., 2013). According to the PA model, both arousal and attention might serve as mechanisms of emotional distortions of duration perception. For the arousal mechanism, high arousal is related to a faster pacemaker (Lui et al., 2011; Smith et al., 2011; Volkinburg and Balsam, 2014; Yoo and Lee, 2015), which causes more pulses to enter the accumulator. As for the attention mechanism, emotional stimuli usually lead to faster detections compared with their neutral counterparts (Hur et al., 2015; Ziaei and Fischer, 2016). This faster detection causes the switch to close earlier (Droit-Volet, 2013a), which causes more pulses to enter the accumulator and consequently, a longer perceived duration. Angrilli et al. (1997) suggested that an arousal mechanism is most influential in the duration perception of high-arousal events. Several studies indicated that at least at quite short durations (i.e., <2 s), the arousal effect plays an important role in the distortion of duration (Angrilli et al., 1997; Droit-Volet et al., 2004; Effron et al., 2006). In the present study, negative and neutral pictures were used as stimuli, and the negative pictures had lower valence and higher arousal than did the neutral pictures. These pictures were presented in range from 400 to 1600 ms. It is therefore reasonable to expect that both the arousal and attention mechanisms made contributions but arousal mechanism contributed more to the emotional distortion of duration perception observed in the nonregulation group.

More interestingly, the results for the reappraisal and suppression groups indicated that emotion regulation modified the emotional distortion of duration perception. When the participants were told to treat the pictures as computer-generated (i.e., the reappraisal group) or to intentionally suppress their own expression of emotion in response to the pictures (i.e., suppression group), we observed no significant differences in the perceived duration between neutral pictures and negative pictures. These results indicated that both reappraisal and suppression can successfully reduce emotional distortions of duration perception. Several previous studies have investigated how to modify the emotional distortion of duration perception (i.e., Effron et al., 2006; Mella et al., 2011; Droit-Volet et al., 2015). As noted in the section “Introduction,” Effron et al. (2006) used a passive, suppression-related method, asking participants to hold a pen in their mouths to suppress their expressions. They found that the perceived duration of emotional faces was no longer than that of neutral faces. Compared with Effron et al. (2006), the suppression method used in the present study does not require any equipment, only prior instruction. This makes it more cognitive and arguably more suitable for use in everyday life (as it is rather inconvenient to hold something in the mouth). In the studies by Droit-Volet et al. (2015) and Mella et al. (2011), different cognitive methods were used to modify the emotional distortion of duration perception. Droit-Volet et al. (2015) used declarative knowledge and found that the overestimation of duration perception was reduced when participants were told that emotional stimuli would have a shortening effect. Mella et al. (2011) used prior instructions – that is, participants were instructed to attend to time, emotion, or both during timing tasks – and found that overestimation of duration perception occurred only when participants attended to emotion. Although Mella et al. (2011), Droit-Volet et al. (2015), and the present study all employed cognitive methods to modify emotional distortion of duration perception, there were some differences between these studies. Droit-Volet et al.’s (2015) method involved directly informing participants of how duration perception is affected by emotion, which suggests that the method modulated duration perception rather than emotion. In contrast, Mella et al.’s (2011) method of instructing participants to attend to or ignore emotion seemed more focused on directing selective attention toward versus away from emotion. In the present study, the emotional distortion of duration perception was modulated by regulating the effect of the emotional stimuli via reappraisal and suppression – in other words, our methods focused on reducing the effects of emotion. In summary, we believe reappraisal and suppression are two new effective methods of modifying emotional distortions of duration perception.

Unexpectedly, we did not observe an underestimated duration perception in the suppression group when compared to the reappraisal group, although the self-report scores of “Did you follow the instructions given to you for judging the duration?” reflected that the suppression group did not perform as well as the reappraisal group. We believe that this difference of self-report scores between suppression and reappraisal groups reflected that suppression is costlier than reappraisal, which has been proved before (e.g., Richards and Gross, 2000; Goldin et al., 2008; Yuan et al., 2014). In other words, suppression made it difficult for participants to follow the instruction to judge the duration. Notwithstanding, in the temporal bisection task, both reappraisal and suppression were successful in reducing the emotional distortion of duration perception, such that the duration perception of the negative and neutral stimuli did not differ. Previous studies have found that individual differences in reappraisal and suppression abilities can affect emotion regulation performance (Hofmann et al., 2009; Yuan et al., 2014). The effort it takes to engage in a particular emotion regulation strategy can also influence such performance (Cai et al., 2016). However, in the present study, participants were randomly assigned to nonregulation, reappraisal, and suppression groups. Furthermore, the self-reported effort to regulate emotion did not differ between the reappraisal group and suppression group. Therefore, the potential influences of emotion regulation ability and effort have been minimized. Consequently, the notion that reappraisal has the same effect as suppression on the emotional distortion of duration perception is credible.

Having clarified the effect of emotion regulation on emotional duration perception, it is now important to consider its underlying mechanisms. According to the PA model, either reducing arousal or decreasing attention bias to emotional events might reduce the emotional distortion of duration perception. Regarding the arousal mechanism, there is evidence from behavioral responses, physiological reactions, and neural activity supporting that both reappraisal and suppression can reduce the arousal of emotional stimuli (Butler et al., 2007; Meyer et al., 2012; Füstös et al., 2013; Ruth et al., 2014; Yuan et al., 2014). This suggests that reduced arousal is a possible mechanism of the observed reduction in the emotional distortion of duration perception. As for attention, studies have shown that both reappraisal and suppression can decrease the attentional bias induced by emotional stimuli (Adam et al., 2014; Vogt and De Houwer, 2014). Accordingly, decreased attention bias could be a mechanism of the results as well. As noted in the second paragraph of discussion, we inferred that the arousal played a dominating role in the emotional distortion of duration perception. It is therefore reasonable to presume that both reduced arousal and decreased attention bias mechanisms are active, but reduced arousal mechanisms are more prominent in the present study. However, this requires further verification.

Several other issues should be clarified. First, in the present study, we only employed Chinese participants. Recent studies have indicated that the efficacy of suppression in dampening negative emotions is culture-specific: People who grew up in East Asian cultures tended to show better suppression performance than did those who grew up in Western countries (Butler et al., 2007; Murata et al., 2013). It has been reported that suppression in Chinese produced beneficial emotion regulation effects in both behavioral and physiological levels (Yuan et al., 2014). Therefore, for Western people, suppression might be a less effective strategy for reducing the emotional distortion of duration perception; further studies should aim to verify this. Second, we used pictures as emotional stimuli. The overestimation of perceived duration has also been found for emotional expressions (Gil and Droit-Volet, 2011; Yoo and Lee, 2015; Ishikawa and Okubo, 2016), sounds (Noulhiane et al., 2007; Zhang et al., 2012), and films (Droit-Volet et al., 2011). However, the effect of duration perception is likely to be different across these stimuli. Thus, the effect of emotion regulation on duration perception should be tested with different types of stimuli. Third, we used instructions to have participants regulate emotion, which is a conscious method of manipulating emotion regulation. However, having participants consciously engage in emotion regulation might lead to cognitive costs (Mauss et al., 2007). Recent studies have indicated that unconscious priming of emotion regulation strategies facilitates realization of regulatory goals without accruing additional costs (e.g., Ding et al., 2015; Chen et al., 2017b). In this regard, future studies should use methods that facilitate more automatic emotion regulation, such as the sentence unscrambling paradigm (Yuan et al., 2015). This might amplify the effect of emotion regulation on the emotional distortion of duration perception by reducing its cognitive costs. Finally, according to the process model of emotion regulation (Gross, 1998), situation selection, situation modification, and attention allocation all have much earlier effects on emotion than do reappraisal and suppression. This is especially true of attention allocation, which has been reported to regulate negative emotion more rapidly than reappraisal (Paul et al., 2013) and protect individuals from developing depressive symptoms during frustrating situations (Li and Yuan, 2018). It can also maintain positive affect and well-being during negative stimulation (Yuan et al., 2012; Meng et al., 2015). As noted above, Mella et al. (2011) successfully used attention allocation to modify emotional distortion of duration perception. Thus, future studies might utilize distraction as a method of optimizing the regulatory effects on emotional distortion.

The findings of present study may have implications for clinical individuals with aberrant distortion of duration perception. For example, recent studies have demonstrated that emotional stimuli result in a larger temporal distortion for anxious individuals (e.g., Bar-Haim et al., 2010; Jusyte et al., 2015; Yoo and Lee, 2015). In line with the findings on healthy individuals, Bar-Haim et al. (2010) suggested that the enhanced arousal in anxious individuals plays a dominant role for aberrant distortion of duration perception in short durations (i.e., <2 s). Therefore, the findings of the present study imply that reappraisal and suppression may be effective methods to reduce aberrant temporal distortion in anxious individuals, and even other clinical individuals who suffer similar aberrant temporal distortion.

In summary, in the present study, Chinese undergraduates were instructed to passively perceive, suppress, and reappraise emotions arising in response to pictures presented for different durations. When passively perceiving the pictures, participants overestimated the duration of negative pictures compared to neutral pictures. However, when either reappraisal or suppression were employed, this overestimation was no longer observed. Therefore, reappraisal and suppression appear to be effective emotion regulation strategies for reducing emotional distortion of duration perception.

## Author Contributions

YT, PL, and XH designed the experiment. YT acquired the data. YT and PL analyzed the data. All authors contributed to the interpretation of the data and approved the final version of the manuscript.

## Conflict of Interest Statement

The authors declare that the research was conducted in the absence of any commercial or financial relationships that could be construed as a potential conflict of interest.
